# Preparation of ICA-loaded mPEG-ICA nanoparticles and their application in the treatment of LPS-induced H9c2 cell damage

**DOI:** 10.1186/s11671-021-03609-9

**Published:** 2021-10-17

**Authors:** Lin Zhou, Zhi Huang, Shanyi Yang, Jiarui Wei, Yan Xu, Lin Hu, Xinrong Guo, Limin Yuan, Zexuan Yuan, Xiaoping Yang, Xiaojun Tao, Qiufang Zhang

**Affiliations:** 1 Department of Geriatrics and General Medicine (QZ) of Affiliated Taihe Hospital, Pharmaology Department (LZ, JW, XG, QZ), School of Basic Medical Science, Hubei University of Medicine, Shiyan, 442000 Hubei China; 2 Key Laboratory of Study and Discovery of Small Targeted Molecules of Hunan Province (ZH, SY, YX, LY, ZY, XY, XT), School of Medicine, Hunan Normal University, Changsha, China; 3 Hubei Key Laboratory of Embryonic Stem Cell Research (XG), Hubei University of Medicine, Shiyan, 442000 Hubei China

**Keywords:** Icariin (ICA), Polyethylene glycol monomethyl ether (mPEG), Fourier infrared spectroscopy, Nuclear magnetic resonance spectroscopy (NMR), Dynamic light scattering (DLS)

## Abstract

Hydrophilic polyethylene glycol monomethyl ether (mPEG) was grafted onto Icariin (ICA) by succinic anhydride to form a polyethylene glycol-Icariin (mPEG-ICA) polymer. The structure of the polymer was characterized by Fourier transform infrared spectroscopy (FT-IR) and nuclear magnetic resonance spectroscopy (NMR). mPEG-ICA nanoparticles loaded with ICA were prepared by physical embedding of ICA by dialysis. The particle size was determined to be (220 ± 13.7) nm, and the ζ potential was (2.30 ± 1.33) mV by dynamic light scattering (DLS). Under a transmission electron microscope (TEM), the nanoparticles were spherical, and the morphology was regular. In the medium with pH 7.4, the drug release rate of mPEG-ICA nanoparticles reached (52.80 ± 1.70)% within 72 h. At pH 6.8, the cumulative drug release of nanoparticles reached (75.66 ± 0.17)% within 48 h. Treatment of the nanoparticles with LPS-treated H9c2 cells maintained cell viability, reduced LDH release and exerted antiapoptotic effects. Moreover, ICA-loaded mPEG-ICA nanoparticles significantly decreased the mRNA expression of the myocardial inflammatory cytokines TNF-α, IL-1β and IL-6M. In conclusion, ICA-loaded mPEG-ICA nanoparticles protected against LPS-induced H9c2 cell injury.

## Introduction

The inflammatory response participates in the whole pathological process of ventricular remodeling and is the main reason for rational remodeling of heart disease after cardiac injury [1, 2]. These proinflammatory cytokines, which include tumor necrosis factor (TNF)-a, IL-1β, IL-6 and IL-18, lead to myocardial injury and long-term pathological remodeling [3]. When cardiomyocytes are damaged, they can also release DAMPs (injury-related molecular models), such as HMGBI, to activate endothelial cells to express chemokine receptors, generate inflammatory factors and induce cardiomyocyte necrosis or apoptosis [4, 5]. In addition, HMGB promotes the accumulation of inflammatory cells, such as macrophages and neutrophils, in the damaged heart through CXCL12/CXCR4, aggravating heart burden and injury [5]. Therefore, blocking the activation of the inflammatory response can be used as an effective strategy to reduce the pathological remodeling of the heart.

Icariin (ICA C_33_H_40_O_15_) is a liposome compound extracted from the Chinese medicinal herba epimedii [6]. Extensive studies have shown that ICA is encouraging as an immunoregulatory, cardioprotective, antioxidant, anti-inflammatory and antiapoptotic agent [7, 8]. Despite the positive properties of ICA, there are various factors that limit its application, including low aqueous solubility, a short half-life, and low oral bioavailability [9]. Nanocarriers have become a new strategy to enhance target-direct efficacy, anti-inflammation and heart protection; in addition, nanocarriers can overcome the disadvantages of ICA [10, 11].

Recently, researchers have focused on drug delivery systems [12]. Various nanocarriers have been developed, such as micelles [13], liposomes [14] and carbon nanotubes [15]. To reduce the mortality and morbidity of cardiovascular disease, it is urgent to design a new nanodosage form loading enough ICA and to have target-directing release properties.

To overcome the limitations of ICA application, we used polyethylene glycol monomethyl ether (mPEG) as a carrier [16, 17]. mPEG is a derivative of polyethylene glycol, which has stable chemical properties and stronger hydrophilicity than PEG [18]. However, because the terminal hydroxyl activity of mPEG is very small, mPEG, as a drug-loaded nanomaterial, must undergo hydroxyl activation. Therefore, we used ICA as the hydrophobic end and carboxyl mPEG formed by the esterification of mPEG and succinic anhydride as the hydrophilic end for chemical connection. The ester bond formed can accelerate cleavage under acidic conditions.

Nanoparticles have unique advantages in the drug delivery of insoluble drugs [19], polymer drugs [20] and gene therapy [21]. Similar to tumor tissue, myocardial inflammatory sites have similar permeability and retention functions (EPRs) [22]. Nanoparticles with a size of approximately 200 nm can penetrate through the interstitial gap to achieve drug enrichment [23]. The mPEG-ICA NPs were prepared in the early stage, confirming their role in myocardial ischemia, but due to the lack of loading ICA, they could not achieve the best effect [24]. Therefore, a new kind of ICA-loaded mPEG-ICA nanoparticle prepared by the combination of chemical synthesis and physical entrapment can not only improve the water solubility and drug loading of ICA but also prolong the sustained release time of nanopreparations in myocardial inflammation and effectively improve the bioavailability of drugs, achieving the best effect of targeted treatment of LPS-induced H9c2 cell damage with ICA. We studied the release of ICA from ICA-loaded mPEG-ICA NPs in pH 7.4 and 6.8 PBS solutions. In addition, to study the effect of mPEG-ICA nanoparticles on myocardial inflammation, we used lipopolysaccharide (LPS) to induce H9C2 cells to establish an external inflammatory model, detected the cell viability, apoptosis rate and mRNA expression of proinflammatory cytokines and then studied the pharmacodynamic effect of ICA-loaded mPEG-ICA nanoparticles in the process of myocardial inflammation.

## Materials and methods

### Experimental instruments

The following were used: electronic balance JA302 (Shanghai Suzhan Metrology instrument Co., Ltd.); rotary evaporator RE52CS-1 (Shanghai Yarong biochemical instrument Factory); digital display constant-temperature magnetic agitator 78HW-1 (Hangzhou instrument Motor Co., Ltd.); electric blast dryer 101-OA (Tianjin Telester instrument Co., Ltd.); Fourier transform infrared spectrometer NEXUS670 (Neliko Co., Ltd.); UV–Vis spectrophotometer (Beijing Leiboteke instrument Co., Ltd.); transmission electron microscopy (TEM Glacios); ultrasonic dispersive extraction instrument water bath oscillator JP-010S (China Jiemeng Co., Ltd.); real-time fluorescence quantitative PCR instrument; multifunction enzyme labeling instrument; inverted microscope (Olympus company) fluorescence microscope (Leica, Heidelberg, Germany); carbon dioxide cell incubator (Thermo company).

### Experimental reagents

The following were used: reagent name purity/specification/production batch number (manufacturer): Icariin (95%/1 g/DL070208 Sahn Chemical Technology Co., Ltd.); lipopolysaccharide (Model 055-100 g/Z06J9Y52452 B5 25 mg Sigma Co., Ltd.); polyethylene glycol monomethyl ether (Analytical Pure/250 g/MKBT7172 V, Sigma Co., Ltd.); dichloromethane (Analytical Pure/500 mL/20171103 Sinopharmaceutical Group Chemical Reagent Co., Ltd.); 4-dimethylaminopyridine (Analytical Pure/100 g/L170741 Shanghai Aladdin Biochemical Technology Co., Ltd.); N-hydroxysuccinimide (Biological Reagent/100 g/RA328L758 Shanghai Ruiyong Biotechnology Co., Ltd.); FastKing one-step method to remove the first strand of genomic cDNA synthesis premixed kit (Tiangen Biotechnology Co., Ltd.); apoptosis detection kit (Biyuntian); SYBR Green fluorescence quantitative kit (QIAGENGmbH company).

### Synthesis of mPEG-COOH

mPEG (5.00 g), succinic anhydride (SA, 0.40 g) and 4-dimethylaminopyridine (molar ratio 1: 1.5/1.5, 0.50 g) were weighed and placed into a 250-mL round-bottom flask. Fifty milliliters of dichloromethane was added, refluxed and stirred at 60 °C for 2 h. The dichloromethane was removed by a rotary evaporator at 35 °C for half a day, and a white solid was obtained, which was kept at room temperature and atmospheric pressure for half a day until no ether residue appeared. The powder was dissolved in 50 mL of secondary distilled water and dialyzed in a 2-kDa dialysis bag for 48 h with the water constantly replaced. Dissolved SA was removed, and undissolved substance was filtered out. Carboxylated mPEG (mPEG-COOH) was obtained by freeze-drying the filtrate and weighing.

### Synthesis of mPEG-icariin polymer

mPEG-COOH(0.37 g), *N*-hydroxy succinimide (0.024 g) and 4-dimethylaminopyridine (0.026 g) were weighed in a 250-mL round-bottom flask, and then 10 mL of dehydrated dimethyl sulfoxide (DMSO) was added as the solvent and stirred at room temperature for half a day to activate the carboxyl group. Then, 100 mg of Icariin standard was placed in a small tube, and 5 mL of DMSO from which the water had been removed was added to dissolve the standard fully. Then, the standard sample was added to the round-bottom flask and stirred for 48 h under the protection of nitrogen gas at room temperature. After continuous dialysis with secondary distilled water, the standard sample did not contain DMSO, and we confirmed the removal of DMSO using UV. Then, the dialyzed material was freeze-dried in a vacuum freeze-dryer for 48 h to obtain a light-yellow product, namely mPEG-ICA polymer.

### Preparation of ICA-loaded mPEG-ICA nanoparticles (NPs)

mPEG-ICA (5 mg) was weighed in a small tube, dissolved in 2 mL of DMSO and then shaken in water at 37 °C for 5 min. Five milligrams of ICA was dissolved in a proper amount of DMSO and put into small beaker, and mPEG-ICA was slowly added and dissolved in the DMSO, and 5 mL of distilled water was added. The solution was then stirred on a magnetic stirrer at room temperature for 15 min. Then, the reactant was dialyzed in a 3500 dialysis bag and dialyzed in water for 24 h, during which the water was constantly changed, the DMSO solvent was dialyzed clean, and the ICA-loaded mPEG-ICA nanoparticles were obtained after filtration.

### Fourier transform infrared spectroscopy

Appropriate amounts of standard ICA, mPEG-COOH and mPEG-ICA polymers were ground into powder in an agate mortar, mixed with appropriate amounts of dry KBr powder and ground into fine powders. After pressing the tablets, the mixture was scanned in a Fourier infrared spectrometer with a scanning range of 4000–4400 cm/mol^−1^, and its infrared spectra were recorded. ICA and mPEG-COOH were used for material characterization, and mPEG-ICA polymer was used for product characterization.

### Nuclear magnetic resonance hydrogen spectrum

ICA, mPEG-COOH and mPEG-ICA polymers were dissolved in deuterated dimethyl sulfoxide (DMSO-d6) and loaded into the sample tube, which was inserted into the rotor. Then, the sample was placed on a 500 MHz nuclear magnetic resonance spectrometer, and the sampling parameters were set.

### UV–Vis spectrum

Icariin standard (4.0 mg) was added to a 25-mL volumetric flask, with methanol added to scale and was then shaken and dissolved until clarified. Then, 0.3 mL, 0.5 mL, 1.0 mL, 1.5 mL, 2.0 mL, and 2.5 mL aliquots of icariin standard solution were accurately measured in 25-mL volumetric flasks, and methanol was added to adjust the volume. The absorbance values of the solutions were then measured on an ultraviolet spectrophotometer at a wavelength of 270 nm (maximum absorption wavelength), with methanol as the blank control. The data were recorded to generate a linear regression.

mPEG-ICA polymer (4.3 mg) was accurately weighed in a 25-mL volumetric flask, with methanol added to scale. The solution was shaken until dissolved and clarified. Then, a 2.0-mL sample solution was accurately measured three times and dissolved in 25-mL volumetric flask, with the volume adjusted with methanol. The absorbance values of the solutions were then measured on an ultraviolet spectrophotometer at a wavelength of 270 nm (maximum absorption wavelength), with methanol as the blank control, and the data were recorded three times, with the average taken. Then, the content of icariin was calculated.

The ICA-loaded mPEG-ICA nanoparticles prepared according to the above method were placed in a 25-mL capacity flask, methanol was added to scale, and the flask was shaken until the solution was dissolved and clarified. Then, a 2.0-mL sample solution was accurately measured three times and dissolved in 25-mL volumetric flask, with the volume adjusted with methanol. The absorbance values of the solutions were then measured on an ultraviolet spectrophotometer at a wavelength of 270 nm (maximum absorption wavelength), with methanol as the blank control, and the data were recorded three times, with the average taken. The data were collected to calculate the average ICA content of ICA-loaded mPEG-ICA nanoparticles.

### Characterization of ICA-loaded mPEG-ICA NPs

#### Dynamic light scattering detection

A dynamic light scattering particle sizer (DLS; The zetasizer 3000hs, Malvern instruments, Malvern, UK) was used to measure the particle size distribution and potential of ICA-loaded mPEG-ICA NPs. The newly prepared mPEG-ICA and ICA-loaded mPEG-ICA NPs were lyophilized and redispersed in distilled water, poured into a colorimetric cup and then placed in a dynamic light scattering particle size analyzer sample pool for detection. Each sample was analyzed three times.

### Observation on the morphology by TEM

The ICA-loaded mPEG-ICA NPs (1.0 mg/mL) solution was dropped on copper mesh with carbon film and filter paper to dry. The grid was placed in the dryer, and 2% (w/w) phosphotungstic acid was added and allowed to naturally dry for 10 min. Then, the morphological characteristics of ICA-loaded mPEG-ICA NPs were observed at an accelerated voltage of 80 kV by transmission electron microscopy.

### Measurement of the stability

The prepared ICA-loaded mPEG-ICA NPs were freeze-dried with 5% mannitol in a vacuum freeze dryer for 48 h, and then the freeze-dried nanoparticles were redissolved in double distilled water to detect the changes in their particle size and PDI value.

### Determination of drug loading and entrapment efficiency

A 1.8-mL ICA-loaded mPEG-ICA NP solution was accurately measured in a 10-mL volumetric flask, 0.2 mL of DMSO was added, and ultrasound was carried out for 2 min. The absorbance of the solution was determined at 270 nm, and the mPEG-ICA polymer with the same solvent was used as a blank. The absorbance of the determined sample was substituted into the standard curve equation, and the Icariin content was calculated. In the DMSO/H_2_O = 1/9 solvent system, the quasi-curve equation of Icariin was measured at 270 nm, and the drug loading (EE%) and entrapment efficiency (LC%) of the nanoparticles were calculated.

- Drug loading (EE%) = drug mass of nanoparticles/total mass of drug-loading nanoparticles × 100%
- Encapsulation rate (LC%) = drug mass in nanoparticles/dosage × 100%.

### Drug release in vitro

Five milliliters of free ICA, mPEG-ICA polymer and ICA-loaded mPEG-ICA NPs was placed in dialysis bags (3500 kDa), which were fastened at both ends an dialyzed in 25 mL of PBS (release medium) under 37 °C and 100 rpm ultrasonic vibration (pH = 7.4). Two milliliters was removed within predetermined periods of time (Tn, *n* = 0, 0.5, 1, 2, 4, 8, 12, 24, 48, and 72 h) and replaced with a fresh solution of the same volume. UV–Vis spectrophotometry was performed to determine the absorbance of the dialysate at 270 nm at the different times. The percentage ratio of ICA release was calculated as described, and three samples were measured to calculate the average drug release. The content of dialysate was determined by the standard curve method, and the release test was repeated 3 times in vitro.

The formula of drug release is *Q*% = (*C*_*n*_ × *V* + *V*_*n*_Σ^*n*^_*t*=0_ *C*_*i*_)/(W_NP_ × LC%), where W is the weight of NP, LC% is the drug loading of nanoparticles, *C*_*n*_ is the sample concentration at Tn, V is the total product of the release medium (25 mL), Vn is the sample weight (2 mL), and *C*_*i*_ is *T*_*i*_ (*i* = 0, 0.5, 1, …., *n* *h*,*V*_0_ = *C*_0_ = 0).

### Release of mPEG-ICA-ICA NPs in different release media

Two milliliters of ICA-loaded mPEG-ICA NP solution was placed into a dialysis bag (4–88 kDa, molecular weight cutoff value) and put into pH 7.4 and pH 6.8 phosphate-buffered saline (PBS release medium, 37 °C, 25 mL). Then, 2 mL of release medium was collected for sampling and replaced with a fresh solution of the same volume at predetermined intervals (Tn, *n* = 0, 0.5, 1, 2, 4, 8, 12, 24, and 48 h). The absorbance of dialysate at 270 nm at the different times was determined by UV–Vis spectrophotometry. The percentage ratio of ICA release was calculated, and three samples were measured to calculate the average drug release.

### Cell testing experiment

H9c2 (rat H9c2), a rat ventricular myoblast line, was purchased from Shanghai Institute of Biochemistry and Cell biology, China. The cells were cultured in DMEM containing 10% fetal bovine serum (Gibco, USA) at 37 °C in a CO_2_-containing atmosphere. H9c2 cells were usually inoculated into a 10-cm culture dish [25]. When the cells grew to approximately 80%, they were subcultured with 0.25% trypsin (Gibco). Then, the cells were seeded in the corresponding culture dishes or 96-well plates for the following studies.

### Cell treatment with LPS or ICA-nanoparticles

H9c2 cells were divided into five groups as follows: (1) control group: using normal culture medium, (2) LPS group: the cells were treated with 10 mg/L LPS for 24 h; (3) ICA group: the cells were treated with 20 μmol/L ICA (1:1000, DMSO) and 10 mg/L LPS for the same duration, (4) mPEG-ICA polymer group: the cells were treated with 20 μmol/L mPEG-ICA polymer and the same final concentration of LPS for the same duration; (5) ICA-loaded mPEG-ICA NPS group: the cells were treated with 20 μmol/L ICA-loaded mPEG-ICA NPs and 10 mg/L LPS, with the other conditions the same as the above group.

### MTT

The cell viability of ICA nanoparticles against LPS-induced cardiomyocyte injury was determined by MTT. Briefly, H9c2 cells were treated with LPS and different ICA nanoparticles for 24 h. After that, the cells were stained with MTT at a final concentration of 0.5 mg/mL for 4 h at 37 °C and then 150 μL of dimethyl sulfoxide (DMSO) was added to dissolve thyroid crystals. Optical density was measured at 570 nm in a microplate reader (*/SpectraMax i3 Molecular Devices, Afghanistan).

### Lactate dehydrogenase (LDH) release

To investigate the effect of nano-ICA on LPS-induced H9c2 damage, we assessed LDH release in the culture medium. Briefly, according to the corresponding treatments for 24 h, the culture medium was collected, and LDH was detected in accordance with the protocol of the LDH Detection Kit (Beyotime Biotechnology). Finally, the OD value was measured at 490 nm with a spectrometer.

### Hoechst 33,258 staining

Hoechst 33,258 staining was used to observe nuclear morphology by fluorescence microscopy. After treatment, H9c2 cells were washed twice with PBS, fixed in 4% paraformaldehyde buffer (Fisher Scientific, Pittsburgh, Pa), rinsed with PBS and stained with Hoechst 33,258 at 37 °C for 15 min [26]. After staining, the nuclear morphologic aspects were immediately observed with a fluorescence microscope (Leica, Heidelberg, Germany). Apoptosis index = number of apoptotic nuclei/total nuclei × 100%.

### TUNEL (terminal dUTP nick-end labeling) assay

For the detection of H9c2 cell DNA integrity, we adopted the TUNEL technique. H9c2 cells were cultured in a 24-well culture plate. After treatment, they were washed 3 times with PBS and fixed with 4% polyformaldehyde for 30 min [27]. Then, 1% Triton X-100 was added for 5 min to increase the cell membrane permeability. Then, the cells were stained with a TUNEL one-step detection kit (Beyotime Biotechnology) according to the instructions, and cell apoptosis was observed with a fluorescence microscope. The apoptosis rate of each group is expressed as the percentage of green fluorescent nuclei (TUNEL-positive) to total nuclei (blue) (the nuclei are stained with DAPI) [28].

### Flow cytometry to detect apoptosis

To further investigate the effects of different ICA nanoparticles on LPS-induced cell apoptosis, we used the Annexin V-FITC/PI double staining method to analyze the apoptotic rate. After H9c2 treatment as described above, cells were harvested and resuspended in 195 μL of binding buffer containing 10 µL of Annexin V-FITC and 5 µL of PI and then incubated for 15 min at room temperature in the dark. After centrifugation at 1500 rpm for 10 min at 4 °C, the staining buffer was aspirated, and the cells were washed twice with PBS and resuspended in 100 µL of PBS. Finally, the samples were measured by flow cytometer.

### Reverse transcription quantitative polymerase chain reaction (RT–qPCR)

RT–qPCR was used to detect the mRNA expression levels of inflammatory markers, including TNF-α, IL-1β and IL-6. After H9c2 cells were treated for 24 h, total RNA was extracted using TRIzol as described previously and measured using a NanoDrop™ One/OneC micro ultraviolet–visible spectrophotometer (Thermo, ND-ONEC-W, USA). cDNA was synthesized using an ExScript RT kit, and amplification was performed on a fluorescent quantitative PCR machine (BIO-RAD CFX96 Touch*) with SYBR Green reagent under the following conditions: 95 °C for 5 min, 95 °C for 30 s, 58 °C for 30 s, and a total of 28 cycles. The primers used in this study are as follows:

Actin: Forward 5′ GCTGTCCCTGTATGCCTCT-3′;
  Reverse 5′-TTGATGTCACGCACGATTT-3′;
TNF-α: Forward 5′TCTATACCACTTCACAAGTCGGA-3′;
  Reverse 5′-GAATTGCCATTGCACAACTCTTT-3′;
IL-1β: Forward 5′GGATGATGACGACCTGCTA 3′;
  Reverse 5′-CACTTGTTGGCTTATGTTCTG3′
IL-6: Forward 5′TGCCTTCTTGGGACTGAT-3′;
  Reverse 5′-CTGGCTTTGTCTTTCTTGTTAT-3′.

The amount of each target gene was normalized to that of the actin gene. The experiments were repeated in triplicate.

## Results and discussion

### FTIR and H^1^ NMR spectra

**Fig. 1:**
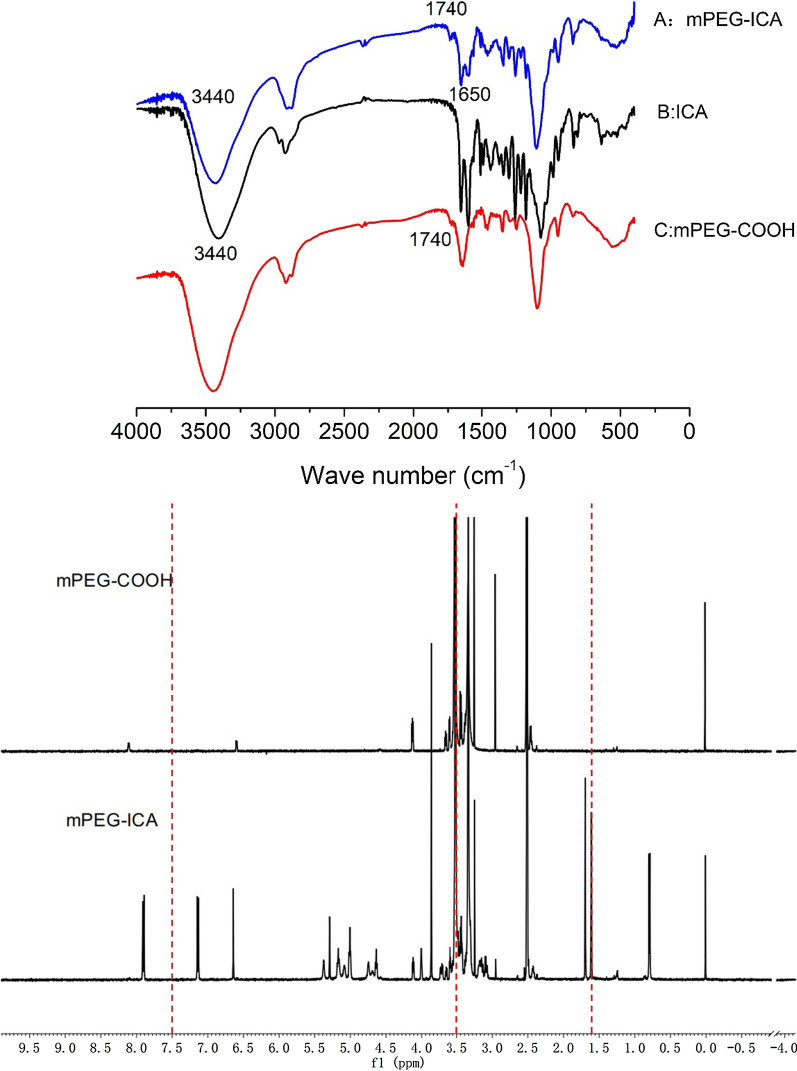
FTIR spectra of mPEG-ICA (**A**), ICA (**B**), and mPEG-COOH (**C**). H^1^ NMR hydrogen spectra for mPEG-COOH and mPEG-ICA

From the mPEG-ICA spectra, the stretching vibration absorption peaks of two ester bonds (–C=O–) were at 1700^−1^ and 1740^−1^ cm. Compared with the mPEG-COOH spectra, there was an excess C=C stretching vibration peak at 1650 cm^−1^, indicating that the esterification synthesis of ICA and mPEG-COOH was successful in forming mPEG-ICA (Fig. 1).

From the mPEG-ICA hydrogen spectrum, 12.6 ppm indicates the ICA phenolic hydroxyl groups due to the presence of –C=O strong absorption electron groups moving to the low field. The benzene ring hydrogen peak at approximately 7.5 ppm and the peak area of 3.5 ppm are very large, judged as the –CH_2_–O– hydrogen peak in the mPEG polymer. A large number of analyses show that the alkyl hydrogen signal is 0–2 ppm. Therefore, it can be concluded that 1.7 ppm is the double bond hydrogen peak in ICA. The characteristic peaks of mPEG-COOH and ICA appear in mPEG-ICA polymers, demonstrating the successful synthesis of mPEG-COOH with ICA (Fig. 1).

### Particle size, zeta potential and TEM

**Fig. 2:**
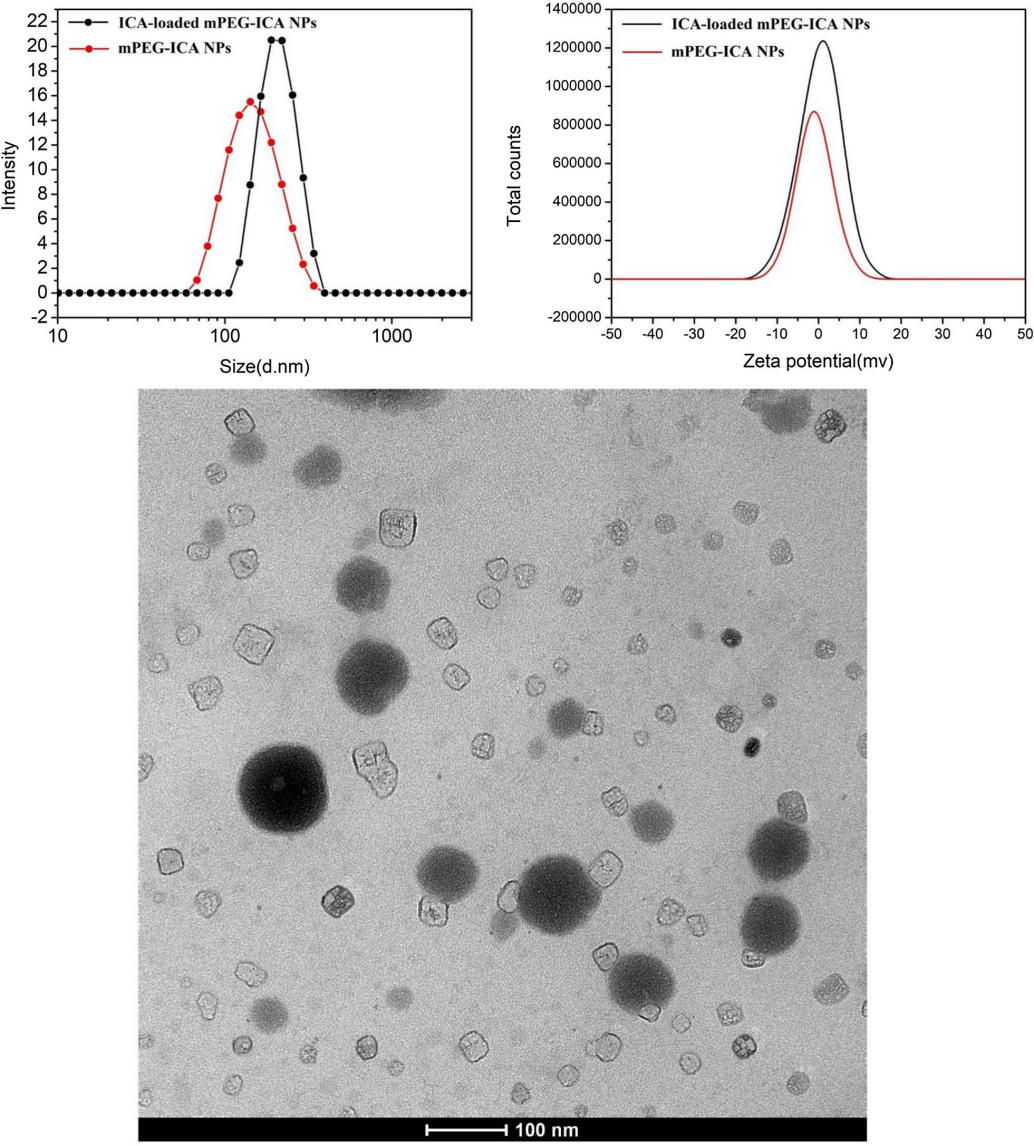
Particle size, zeta potential and TEM of ICA-loaded mPEG-ICA NPs

The average particle size of the mPEG-ICA nanoparticles is approximately (145.0 ± 15.2) nm, and the dispersion index (PDI) value of the polymer is (0.277 ± 0.00). The particle size of ICA-loaded mPEG-ICA nanoparticles increased slightly, to approximately (220.0 ± 13.7) nm, and the PDI was (0.119 ± 0.00). The smaller the PDI is, the more uniform the distribution of ICA-loaded mPEG-ICA NPs. The zeta potential of the mPEG-ICA nanoparticles was (0.439 ± 0.258) mV, and the zeta potential of ICA-loaded mPEG-ICA NPs was (2.30 ± 1.33) mV. Transmission electron microscopy revealed that the ICA-loaded mPEG-ICA nanoparticles were spherical, regular in morphology and relatively uniform in size (Fig. 2).

### Drug loading and encapsulation efficiencies

The ICA content in the ICA-loaded mPEG-ICA NPs can be calculated by ultraviolet spectrophotometry. The standard curve of ICA concentration was established, and the ICA content in the polymer was obtained by calculating the drug concentration based on the standard curve. According to the calculations, based on the mPEG-ICA polymer absorbance (1.2397 ± 0.1024), the content of ICA was (0.4132 ± 0.0359) mg/mL, with ICA-loaded mPEG-ICA nanoparticle absorbance (1.6289 ± 0.0923), and the content of ICA was (0.5496 ± 0.3234) mg/mL. Calculated according to the formula in the method, the drug loading of mPEG-ICA polymer was (16.5 ± 0.014) %, and the encapsulation rate was (41.3 ± 0.036)%; drug loading content of the ICA-loaded mPEG-ICA NPs was (21.9 ± 0.013)%, and the encapsulation rate was (54.9 ± 0.032) % (Table 1).

**Table 1:** Comparison of mPEG-ICA NPs and ICA-loaded mPEG-ICANPs

Name	Size (nm)	PDI	Potential (mV)	Drug delivery rate (%)	Encapsulation rate (%)
mPEG-ICA NPs	(145.0 ± 15.2)	(0.277 ± 0.0)	(0.44 ± 0.26)	(16.5 ± 0.014)%	(41.3 ± 0.036)%
ICA-loaded mPEG-ICA NPs	(220.0 ± 13.7)	(0.119 ± 0.0)	(2.30 ± 1.330)	(21.9 ± 0.013)%	(54.9 ± 0.032)%

### Stability determination

**Fig. 3:**
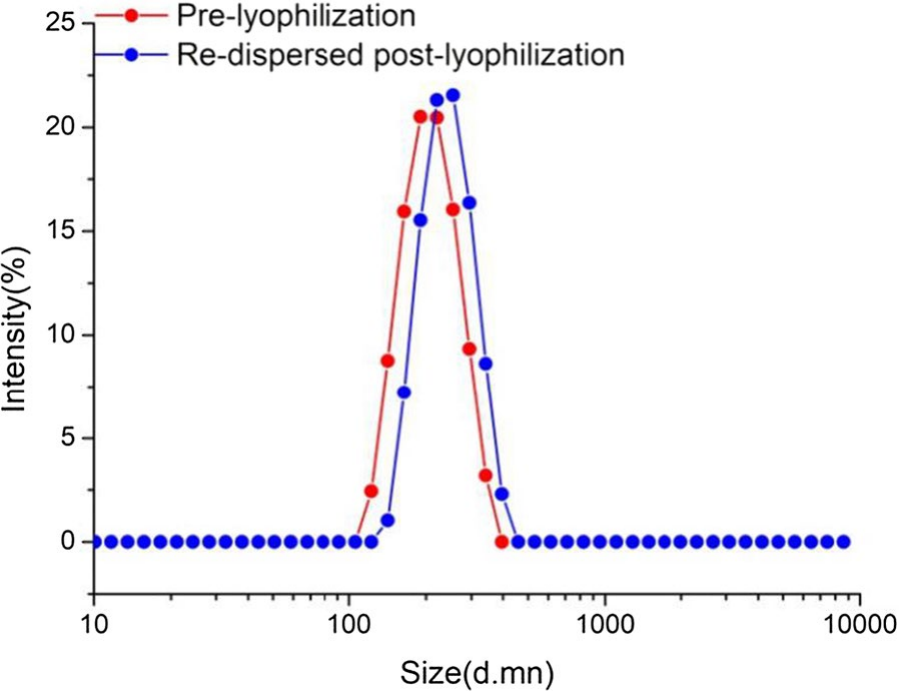
Stability of ICA-loaded mPEG-ICA NPs

The particle size of ICA-loaded mPEG-ICA nanoparticles prepared by the dialysis method was (220.0 ± 13.7) nm, and the PDI was (0.119 ± 0.00). After freeze-drying in 5% mannitol for 48 h, the nanoparticles can be redissolved in water to form stable nanoparticles. The particle size was 255 nm, and the PDI was (0.326 ± 0.00), which was slightly larger than that of dialysis nanoparticles (Fig. 3).

### mPEG-ICA-ICA drug release in vitro

**Fig. 4:**
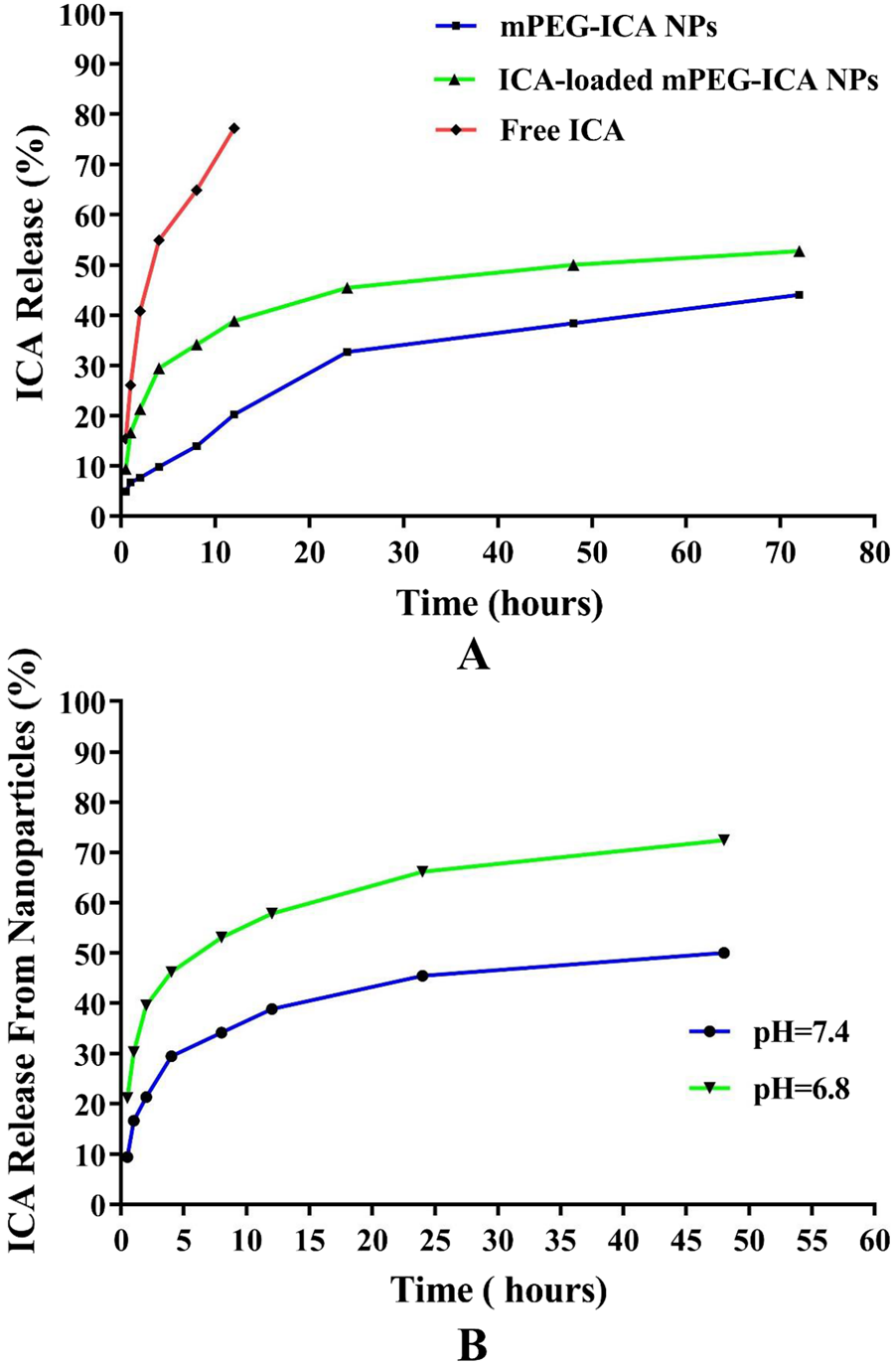
**A** ICA release from ICA-loaded mPEG-ICA NPs in PBS at pH 7.4. **B** ICA release from ICA-loaded mPEG-ICA NPs in PBS at pH 7.4 and pH 6.8 at 37 °C in vitro

In Fig. 4, ICA release largely depends on the pH of the release medium. Within phosphate-buffered saline (PBS) release medium at pH 7.4, free icariin was released (77.21 ± 0.15)% within 12 h and released completely. Because the mPEG-ICA NPs loaded with icariin drugs can increase the release of nanoparticles, drug release of mPE-ICA nanoparticles released to (44.08 ± 0.12)% within 72 h, and ICA-loaded mPEG-ICA nanoparticles reached (52.80 ± 1.70)%. ICA-loaded mPEG-ICA nanoparticles were placed in a buffer solution of pH 6.8, and we found that the nanoparticle-mediated release of drugs reached (75.66 ± 0.17)% within 48 h. This is likely because some ICA-loaded mPEG-ICA NPs had depolymerized NPs or mPEG-ICA molecules break in NPs. These observations indicated that the release of ICA was more favorable under acidic conditions of pH 6.8. It also indicated that the release characteristics of ICA-loaded mPEG-ICA nanoparticles were beneficial to the local treatment of myocardial inflammatory injury, because local lesions lead to a focus with a weakly acidic environment.

### H9C2 cell viability and lactate dehydrogenase release

**Fig. 5:**
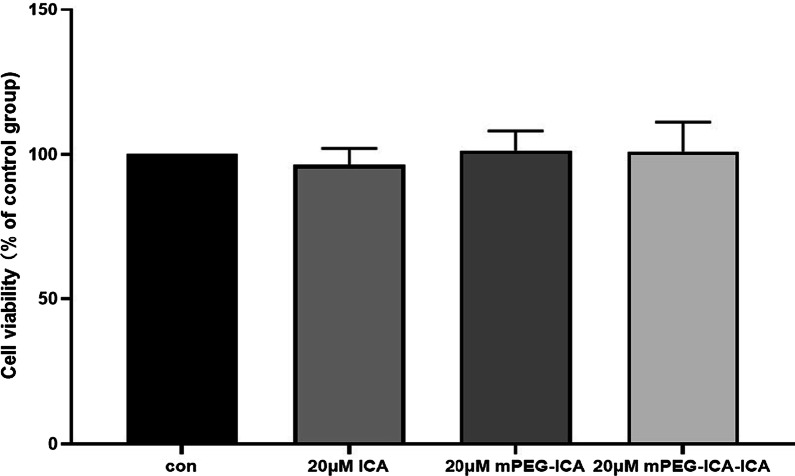
Evaluation of the cytotoxicity of ICA nanoparticles on H9c2 cells. Data are expressed as the mean ± SEM of three separate experiments

**Fig. 6:**
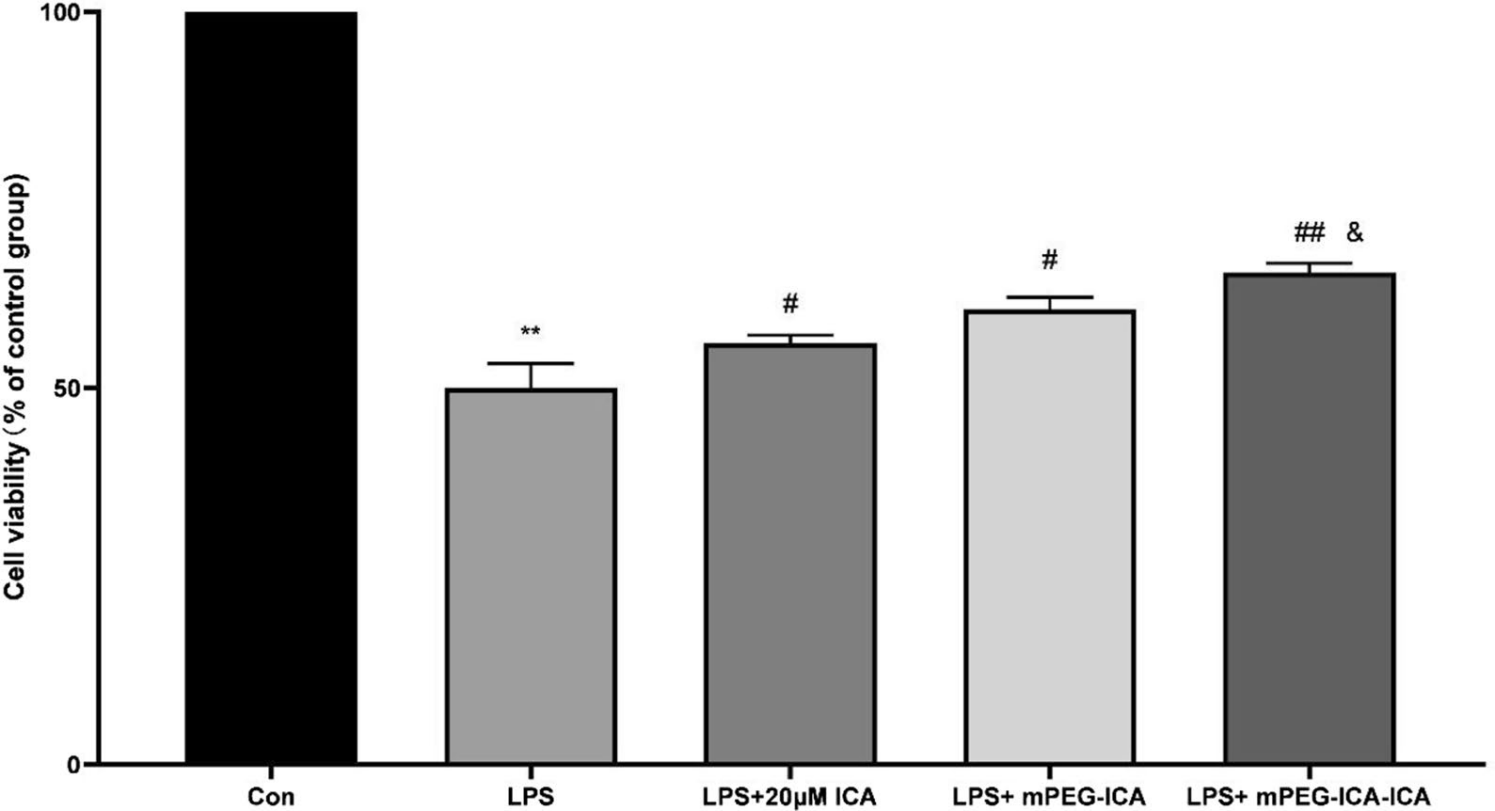
Evaluation of the effects of ICA nanoparticles on LPS-induced H9c2 cell injury. Cell viability in different groups was measured by MTT assay. Data are the mean ± SME. (**P < 0.01 vs. con; # *P* < 0.05 and, ## *P* < 0.01 vs. LPS; & *P* < 0.05 vs*.* LPS + mPEG-ICA)

First, to observe the cytotoxicity of ICA-loaded mPEG-ICA nanoparticles, we added free drug (20 µM ICA), mPEG-ICA NPs and ICA-loaded mPEG-ICA NPs to H9c2 cells, and the MTT results showed no marked cytotoxicity of the nanoparticles (Fig. 5). Then, we further explored whether they could protect H9c2 cells against LPS-induced injury (Fig. 6). After LPS treatment for 24 h, cell viability was reduced to (50.0 ± 3.22)% (control group 100%), where treatment with ICA, mPEG-ICA NPs and ICA-loaded mPEG-ICA NPs significantly increased the viability of H9c2 cells by (55.94 ± 1.06)%, (60.97 ± 2.615)%, and (65.36 ± 1.214)%, respectively (*P* < 0.05).

**Fig. 7:**
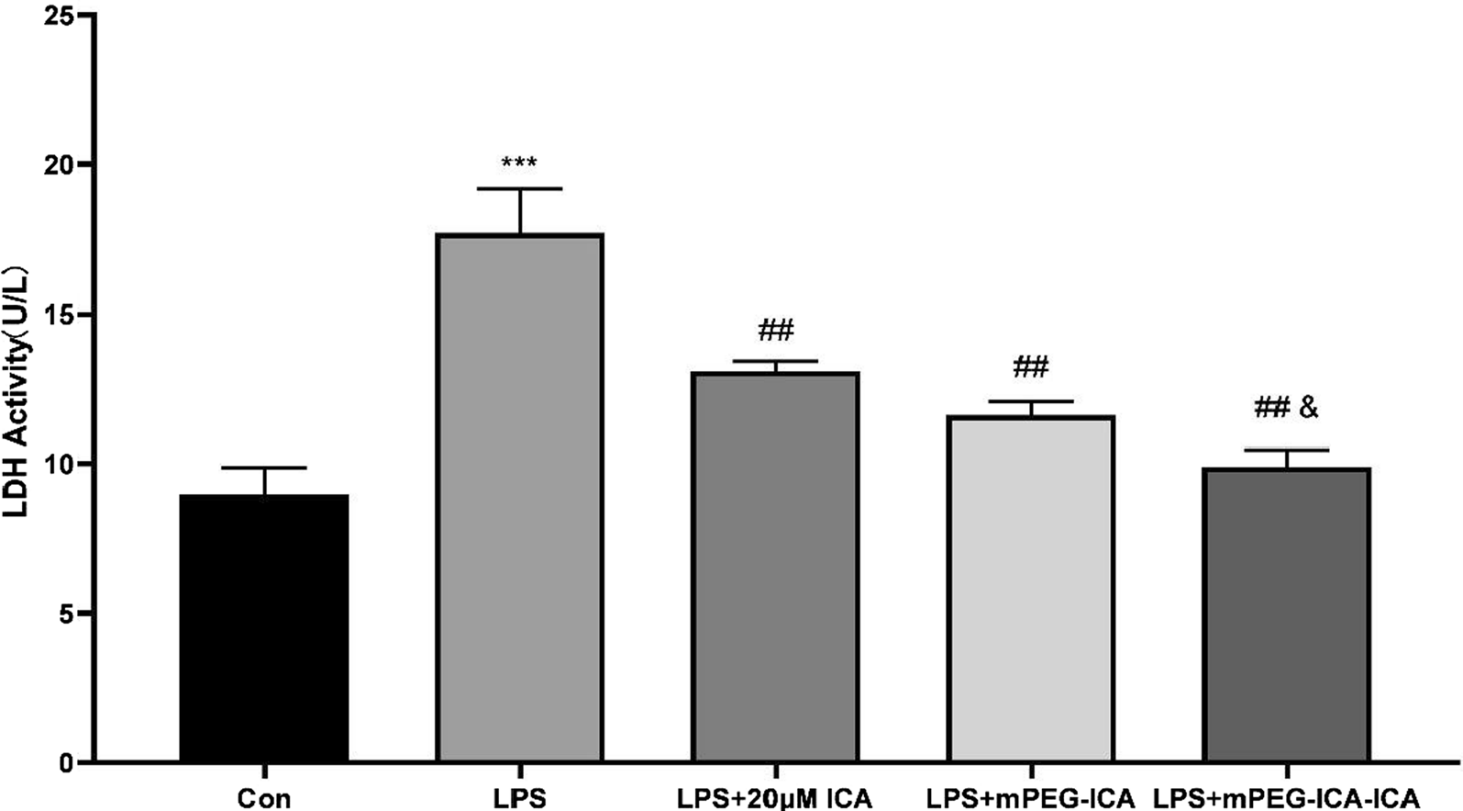
The effect of ICA nanoparticles on the LDH level induced by LPS in H9c2 cells. Data are the mean ± SD. ***P < 0.01 versus con; ##*P* < 0.01 versus LPS treatment group; *& *P* < 0.05 versus LPS + mPEG-ICA group

In addition, LDH levels are a marker of cell damage; therefore, we measured the release level of LDH in the culture medium (Fig. 7). LDH levels were increased in the LPS group compared with the normal culture group. Treatment with ICA, mPEG-ICA NPs or ICA-loaded mPEG-ICA NPs reduced the LDH level increase induced by LPS, especially in the ICA-loaded mPEG-ICA NP group. These results showed that ICA nanoparticles could protect cells from LPS.

### Cell apoptosis induced by LPS

**Fig. 8:**
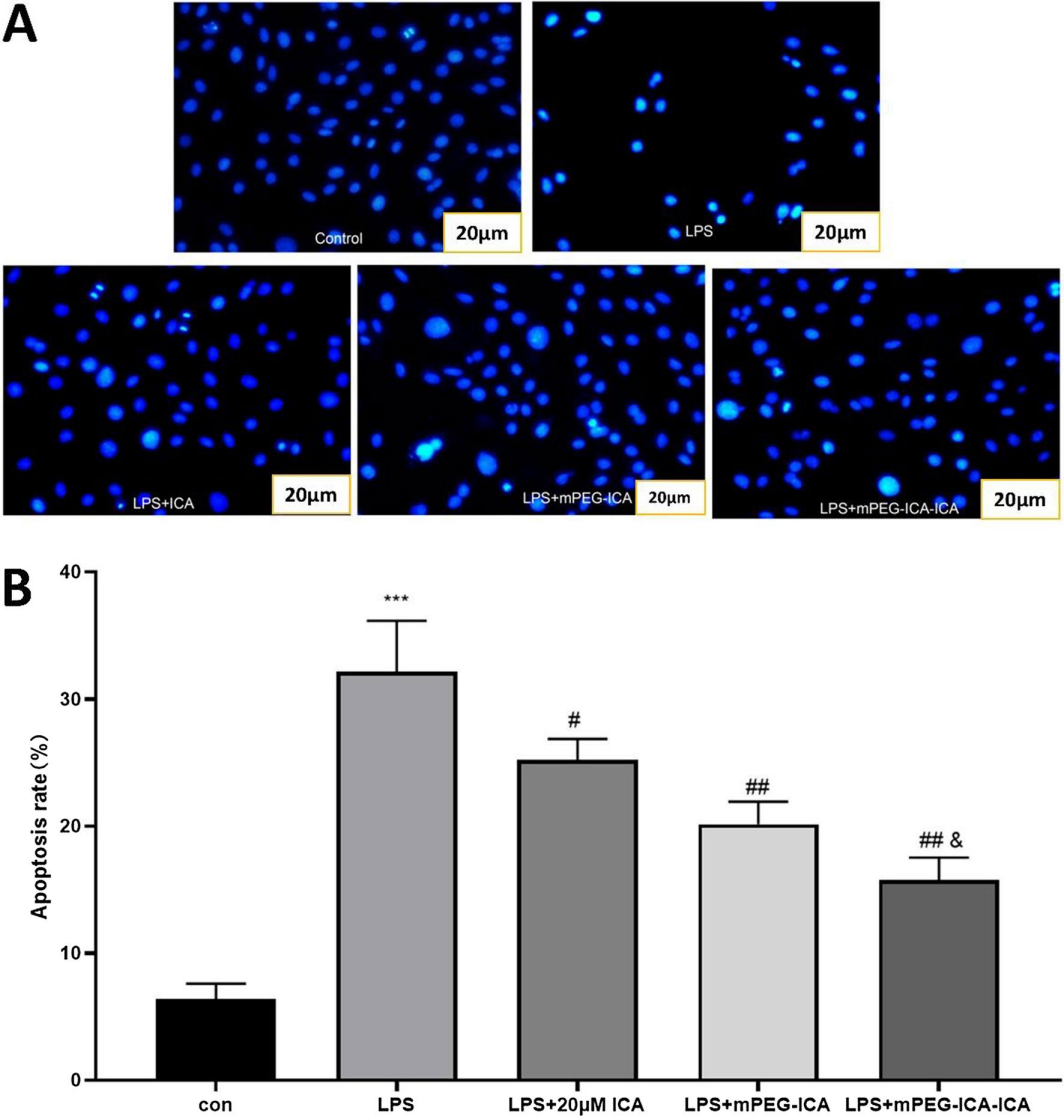
Detection of apoptosis by Hoechst 33,258 staining. **A** Nuclear morphology in cells stained with Hoechst 33,258. **B** Quantitative analysis of the apoptosis rate by Hoechst 33,258 staining. Data are the mean ± SEM (***P < 0.005 vs. con; #*P* < 0.05 vs. LPS; &*P* < 0.05 vs. LPS + mPEG-ICA)

**Fig. 9:**
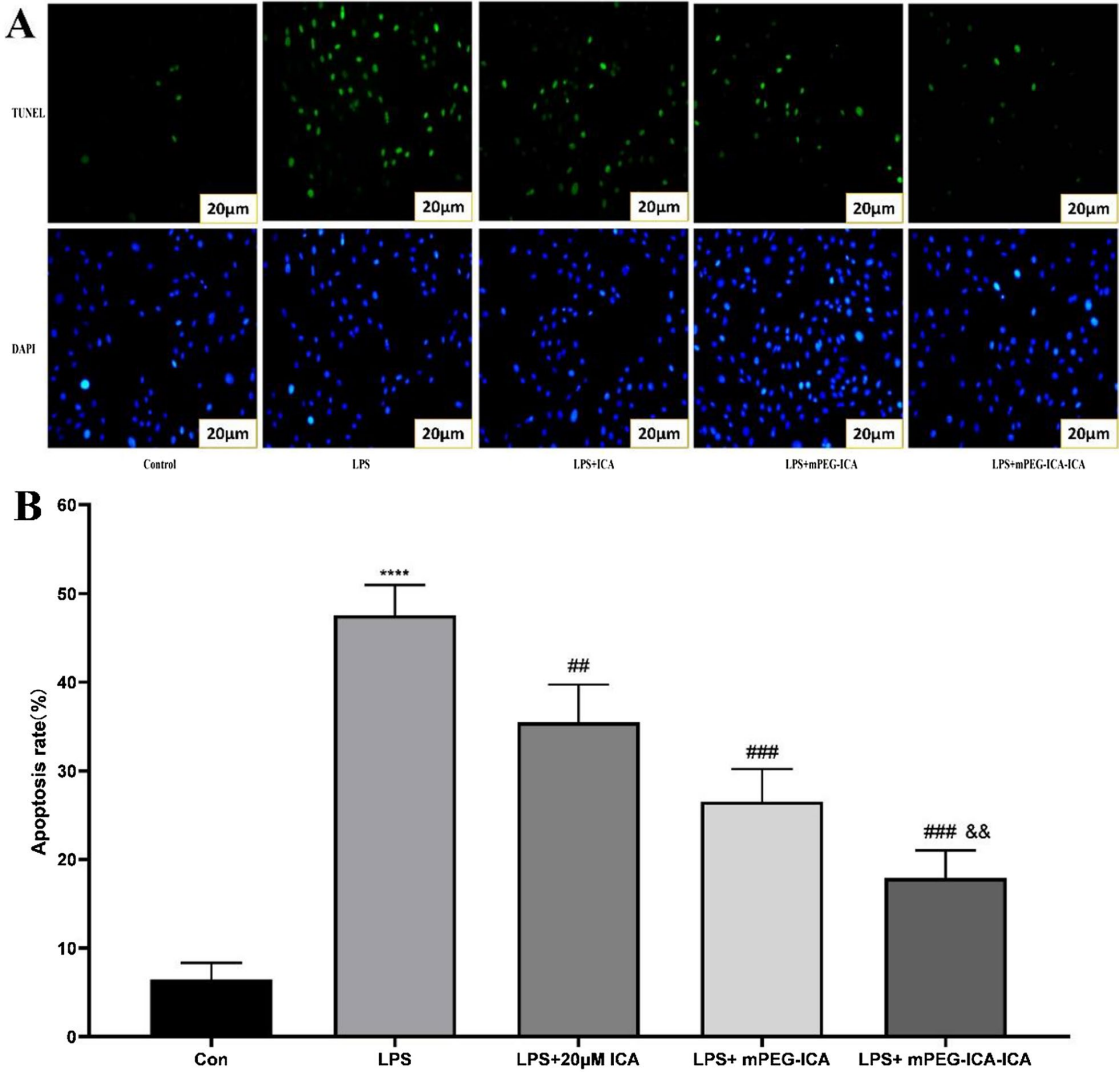
Detection of apoptosis by TUNEL. **A** Cells with TUNEL and DAPI staining; **B** apoptosis rate. Data are the mean ± SEM (****P < 0.0001 vs*.* con; ###*P* < 0.005 vs. LPS; &&*P* < 0.01 vs. LPS + mPEG-ICA)

**Fig. 10:**
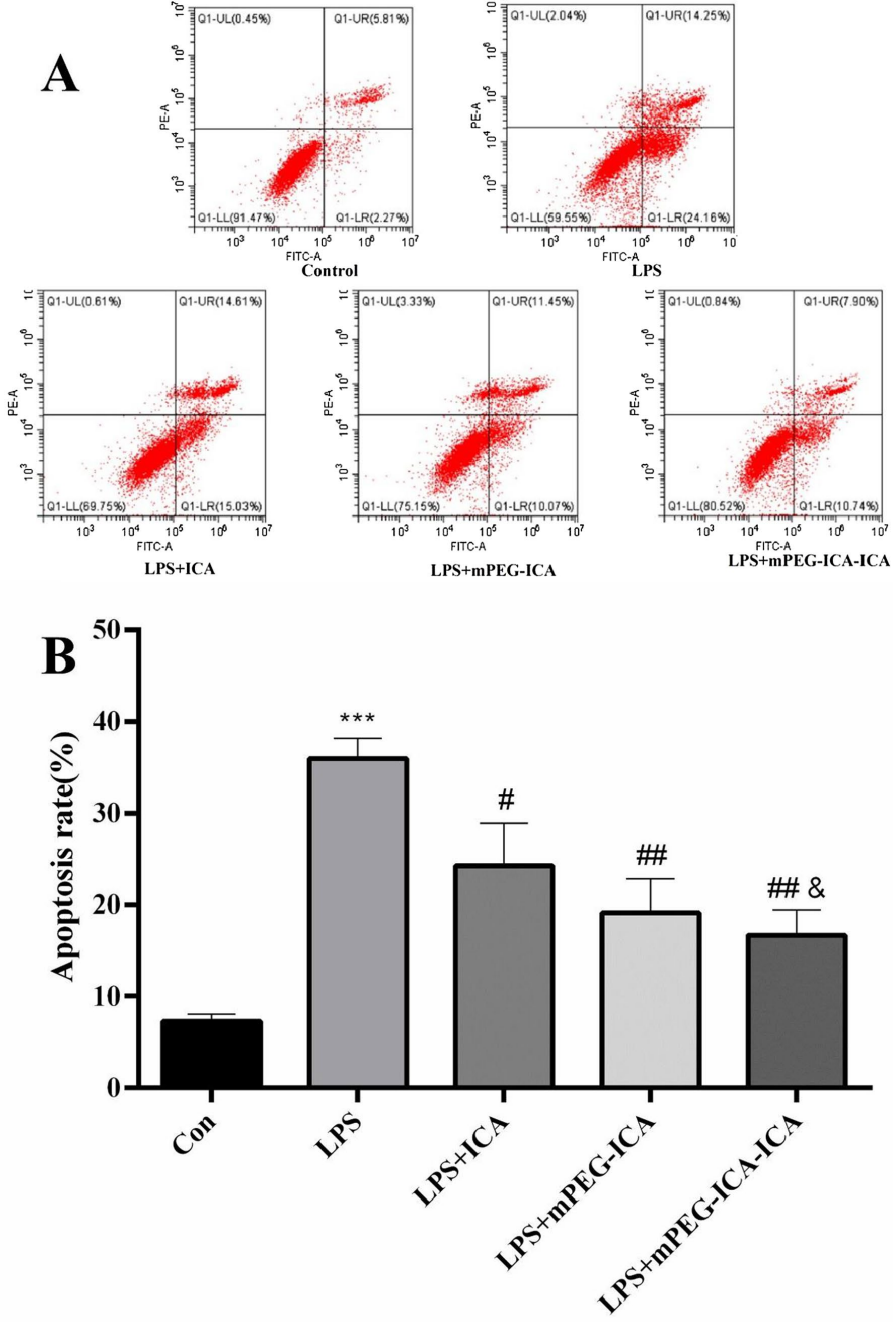
**A**. Apoptotic rate detected by flow cytometry. H9c2 cells were stained by Annexin V/PI double staining. Q1-LL cells were Annexin V/PI double-negative stained, indicating viable cells; Q1-LR cells were Annexin V-positive and PI-negative stained, indicating early apoptosis; Q1-UR cells were Annexin V/PI double-positive stained, indicating late apoptosis. **B**. Apoptotic rate (%). Data are the mean ± SME. (***P < 0.005 vs. con; #*P* < 0.05 and ##P < 0.01 vs. LPS; &*P* < 0.05 vs. LPS + mPEG-ICA)

First, nuclear morphology was detected by Hoechst 33,258 staining (Fig. 8). Nuclear chromatin exhibited condensation, breakage and fragmentation after LPS damage in the LPS group. However, for ICA-loaded mPEG-ICA NP group, the number of apoptotic cells obviously decreased**.** Moreover, we used TUNEL staining to observe cellular DNA breaks or DNA damage (Fig. 9). The number of apoptotic nuclei in the LPS-induced group was (47.57 ± 3.41)% *(P *˂ 0.0001) compared to (6.47 ± 1.87)% in the normal culture group. After treatment with ICA-loaded mPEG-ICA NPs, mPEG-ICA NPs and ICA, the apoptosis rates decreased to (17.92 ± 3.12)% and (26.54 ± 3.68)% and (35.49 ± 4.25)%, respectively, and ICA-loaded mPEG-ICA NPs significantly reduced the apoptosis rate compared with mPEG-ICA NPs. In addition, flow cytometric analysis in LPS-induced H9c2 cells showed that (35.93 ± 2.23)% of the cells were in the early and late apoptotic stages (Fig. 10). After treatment with ICA-loaded mPEG-ICA NPs or mPEG-ICA NPs, the total apoptotic rates were reduced to (16.70 ± 2.77)% and (19.15 ± 3.67)%, respectively. These results were in line with the Hoechst 33,258 staining and TUNEL staining findings and indicated that ICA-loaded mPEG-ICA NPs largely prevented the cells from undergoing apoptosis.

### Inflammatory cytokine mRNA in LPS-induced H9c2 cells

**Fig. 11:**
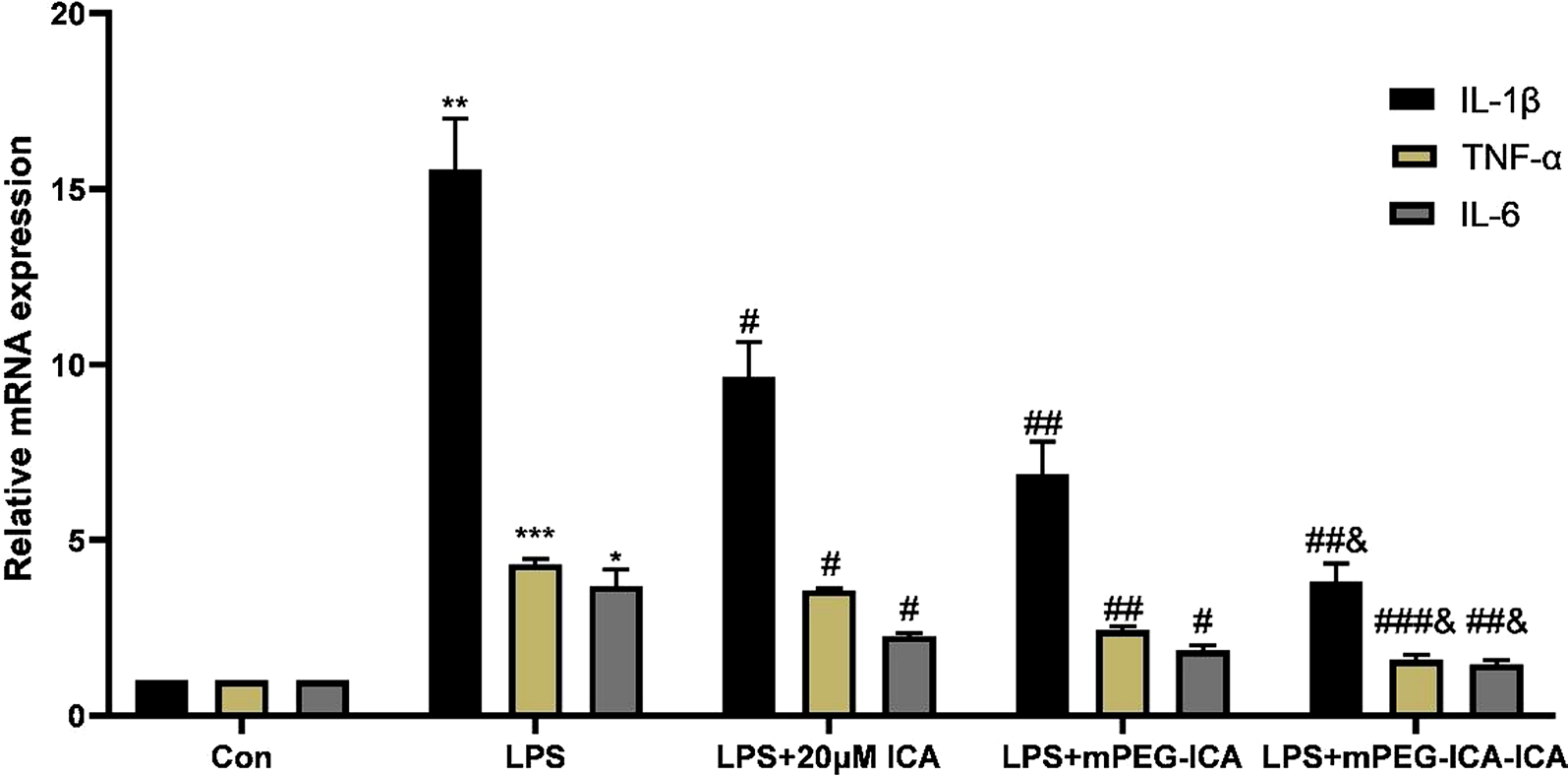
Effects of ICA-loaded mPEG-ICA NPs on the mRNA expression levels of IL-1β, TNF-α and IL-6 in LPS-induced H9c2 cells. Effects of ICA-loaded mPEG-ICA NPs on the mRNA IL-1β in the LPS induced H9c2 cells. (**P < 0.01 compared with con; #*P* < 0.05 compared with LPS; & *P* < 0.05 compared with LPS + mPEG-ICA.) (2) Effects of ICA-loaded mPEG-ICA NPs on the mRNA TNF-α. (***P < 0.005 vs. con; #*P* < 0.05 vs. LPS group; &*P* < 0.05 vs. LPS + mPEG-ICA) (3) Effects of ICA-loaded mPEG-ICA NPs on mRNA IL-6. Data are the mean ± SEM (* *P* < 0.05 vs. con; #*P* < 0.05 vs. LPS; & *P* < 0.05 vs. LPS + mPEG-ICA)

Next, we evaluated the effect of ICA-loaded mPEG-ICA NPs on the mRNA expression of TNF-α, IL-1β and IL-6 in LPS-induced H9c2 cells. After LPS treatment for 24 h in H9c2 cells, the mRNA levels of TNF-α, IL-1β and IL-6 were increased. These increases were attenuated by ICA-loaded mPEG-ICA NPs, mPEG-ICA NPs and ICA treatment, and ICA-loaded mPEG-ICA NPs treatment markedly lowed TNF-α, IL-1β and IL-6 mRNA expression (Fig. 11).

In this study, we analyzed the suitability of polymer nanoparticles to load ICA for the treatment of LPS-induced H9c2 cell damage. The synthesis method is highly feasible and has many advantages. First, mPEG is nontoxic and has good biocompatibility [29]. In this study, a chemical bonding method was used to assemble mPEG and ICA into an mPEG-ICA polymer, and then dialysis was used to physically wrap the ICA and form stable nanoparticles [30]. Nanoparticles can significantly improve the water solubility of ICA and can realize the long circulation of nanoparticles in the body [31, 32]. Compared with mPEG-ICA nanoparticles, ICA-loaded mPEG-ICA nanoparticles have many significant advantages. First, their PDI value is smaller, which proves that the particle size distribution is more uniform [33]. Second, the drug loading and encapsulation efficiencies of ICA-loaded mPEG-ICA NPs are both higher than those of mPEG-ICA NPs, making it easier to reach the effective drug concentration of ICA [34]. In a pH 6.8 solution, nanoparticles release more drugs, and an acidic environment may destroy the self-assembly force of nanoparticles and accelerate the cleavage of ester bonds, resulting in faster release of the drug into the medium and effective enrichment of the drug in the inflammation site [35, 36]. Huang et al. used LPS-induced osteoblasts to validate the model to explore the anti-inflammatory mechanism of Icariin. They found that Icariin can significantly upregulate the expression of BMP-2 and Runx2 in LPS-induced osteoblasts, thereby achieving effective therapeutic effects [37]. A study has shown that ICA can inhibit the inflammatory response of chondrocytes induced by LPS and reduce collagen formation [38]. In addition, ICA can also inhibit the caspase-1 signaling pathway mediated by the NLRP3 inflammasome and reduce LPS-induced sagging [39]. However, LPS-induced cells cannot effectively absorb ICA. Making ICA nanoparticles can solve this problem well. It may be that ICA is loaded by nanocarriers, greatly increasing its water solubility and allowing it to enter cells through free diffusion. At the same time, studies have shown that nanoparticles can promote cell endocytosis and tissue cell efflux and significantly improve the bioavailability of ICA [40, 41].

The results of the MTT assay and lactate dehydrogenase release experiments showed that ICA-loaded mPEG-ICA NPs could effectively alleviate the cell damage induced by LPS, and ICA-loaded mPEG-ICA NPs could release ICA from the particles. Furthermore, compared with the mPEG-ICA NPs, ICA-loaded mPEG-ICA NPs could load more ICA and release more easily. We also found that these nanoparticles markedly decreased apoptosis induced by LPS to alleviate the inflammatory response in H9c2 cells. Moreover, the antiapoptotic effect of ICA-loaded mPEG-ICA NPs was better than that of mPEG-ICA NPs and ICA. These results indicated that ICA nanoparticles could protect cardiomyocytes from inflammatory injury.

Inflammatory cytokines are the key factors involved in the development and progression of cardiovascular disease and are markers of the inflammatory response [42, 43]. Some researchers have found that TNF-α, IL-1β and IL-6 were closely related to cardiac function. The addition of these inflammatory cytokines to isolated cardiomyocytes resulted in abnormalities in cardiac function and promoted cardiomyocyte loss [44]. Our RT–qPCR results showed that ICA-loaded mPEG-ICA NPs could significantly inhibit the mRNA expression of TNF-α, IL-1β and IL-6. Therefore, these results implied that the protective effects of ICA-loaded mPEG-ICA NPs may occur through controlling inflammatory cytokines.

## Conclusion

ICA-loaded mPEG-ICA NPs are a new type of nanomedicine successfully synthesized through nanotechnology (a combination of chemical synthesis and physical embedding). This process not only converts the hydrophobic drug ICA into water-soluble nanomedicine but also successfully improves the load capacity of ICA. In the weakly acidic solution, the ICA release from ICA-loaded mPEG-ICA NPs was greater than that of the neutral solution, indicating that nanoparticles are meaningful for the treatment of inflammatory cardiovascular diseases. Treatment with ICA nanoparticles effectively protected against LPS-induced H9c2 damage, promoted cell viability and inhibited cell apoptosis. Further experiments demonstrated that the new ICA-loaded mPEG-ICA NPs could release inflammatory cytokine production.

## Data Availability

Not applicable.

## Abbreviations

mPEG: Polyethylene glycol monomethyl ether
ICA: Icariin
FT-IR: Fourier transform infrared spectroscopy
NMR: Nuclear magnetic resonance spectroscopy
DLS: Dynamic light scattering
TEM: Transmission electron microscope
TNF: Tumor necrosis factor
EPRs: Permeability and retention functions
SA: Succinic anhydride
DMSO: Dehydrated dimethyl sulfoxide
